# Myricitrin exhibits anti-atherosclerotic and anti-hyperlipidemic effects in diet-induced hypercholesterolemic rats

**DOI:** 10.1186/s13568-019-0924-0

**Published:** 2019-12-21

**Authors:** Jing Gao, Cuicui Liu, Heping Zhang, Zhen Sun, Rongmei Wang

**Affiliations:** 1 Department of Cardiovascular Surgery, Affiliated Hospital of Qingdao University, Qingdao, 266000 China; 2 Department of Anesthesia, Affiliated Hospital of Qingdao University, No. 16 Jiangsu Road, Shinan District, Qingdao, 266000 China

**Keywords:** Myricitrin, Atherosclerosis, Rats, Anatioxidants, Cholesterol

## Abstract

The present study investigated the anti-atherosclerotic potential of myricitrin in hypercholesterolemic rats. Rats were divided into the following groups: sham (standard food), control [1% high-cholesterol diet (HCD)], 1 μM myricitrin + 1% HCD, 10 μM myricitrin + 1% HCD, 100 μM myricitrin + 1% HCD, and the positive control (10 mg/kg body weight atorvastatin). The dose was given to rats via oral gavage for 45 consecutive days. Feeding of rats with 1% HCD caused substantial increases in the levels of LDL, cholesterol, and triglycerides (TG), while high-density lipoprotein (HDL) was reduced. However, rats supplemented with myricitrin had reduced levels of cholesterol, LDL, and TG to near-normal levels, whereas HDL was increased. Catalase, superoxide dismutase (SOD), glutathione peroxidase (Gpx), and reduced glutathione (GSH) levels were substantially reduced in the HCD-fed rats compared with sham rats. However, the rats supplemented with 100 μM myricitrin showed > 50% increases in these levels. Lipid peroxidation and reactive oxygen species (ROS) levels were reduced following myricitrin treatment. The aortic cell wall area was significantly increased by 14.5% in HCD-fed rats. However, rats supplemented with 1, 10, and 100 μM myricitrin showed significant reductions in the aortic cell wall area of 2.3%, 4%, and 27.5%, respectively. This is the first report of the anti-atherosclerotic and hypolipidemic effects of myricitrin in hypercholesterolemic rats. Myricitrin decreased the level of total serum cholesterol and the role of aortic atherosclerosis in hypercholesterolemic rats.

## Introduction

Atherosclerosis is a well-known multifactorial and progressive disease contributed by oxidation of low‐density lipoprotein (LDL), endothelial dysfunction, and oxidative stress (Rashidi et al. 2017). Cardiovascular diseases account for > 30% of deaths worldwide (Bader 2010). Atherosclerosis is central to cardiovascular diseases, including unstable angina, sudden cardiac death, stroke, myocardial infarction, and peripheral thrombosis (Xu et al. 2018). A previous study showed that the complex disease process of atherosclerosis was due to the interaction between inflammation and perturbations (van Diepen et al. 2013). Another study showed that inflammation and dyslipidemia are the primary mechanisms involved in the pathophysiology of atherosclerosis (Singh et al. 2002). An additional study showed that increased levels of total cholesterol, plasma triglycerides (TGs), and LDL cholesterol are characteristic features of hyperlipidemia (Tonstad and Despres 2011). Obesity, dyslipidemia, and hypertension trigger several inflammatory events, which cause foam cell formation and monocyte recruitment to atherosclerotic lesions (Libby et al. 2002). Furthermore, the interactions between inflammatory events and lipid metabolism aggravate the development of atherosclerosis (van Diepen et al. 2013).

Myricitrin is a well-known flavonoid obtained from few plants such as *Eugenia* uniflora, Pouteria gender and Manilkara zapota (Asano et al. 2007), and previous studies have shown the anxiolytic and antinociceptive effects of myricitrin (Pereira et al. 2011). Sun et al. (2013) have reported the protective effect of myricitrin against the vascular endothelial cell apoptosis induced by oxidative stress. Inhibitory effect of myricitrin against the early atherosclerosis and endothelial damage induced by oxidative stress in ApoE^−/−^ mice. Qin et al. (2015) showed a protective effect of myricitrin against oxidized LDL-induced endothelial cell apoptosis and its potential therapeutic activity against cardiovascular diseases. Based on these earlier reports, the present study investigated the anti-hyperlipidemic and anti-atherosclerotic potential of myricitrin in diet-induced hypercholesterolemic rats.

## Materials and methods

### Rats and diet

Male albino rats were obtained from the animal house of the Affiliated Hospital of Qingdao University, Qingdao, China. Each rat weighed 190–210 g. The rats weighed 190–210 g and rats were kept in standard rat polypropylene cages (435 × 290 × 150 mm; six rats per cage) and maintained under standard conditions of 12 h light/12 h dark at a relative humidity of 60 ± 5% and temperature of 25 ± 0.5 °C with food and water provided ad libitum. All animal experiments complied with the guidelines of the Department of Anesthesia, Affiliated Hospital of Qingdao University, No. 16 Jiangsu Road, Shinan District, Qingdao, China, which approved all animal care and experimental protocols. Animal surgery was conducted under anesthesia, and every effort was carefully made to reduce suffering. All rats were maintained under appropriate conditions according to ethical standards for animal welfare. A high-cholesterol diet (HCD) was prepared by the addition of 96 g standard laboratory food to 1 g cholesterol and 3 g corn oil (Huseini et al. 2015).

### Experimental groups

The rats were divided into the following groups: sham (standard food), control (1% HCD), 1 μM myricitrin (91255, CAS Number 17912-87-7, Sigma-Aldrich, Shangai) + 1% HCD, 10 μM myricitrin + 1% HCD, 100 μM myricitrin + 1% HCD, and the positive control (10 mg/kg body weight atorvastatin). Myricitrin was dissolved in saline and control rats were given equal volume of saline. Each group contained six rats. The dose was given to the rats via oral gavage for 45 consecutive days.

### Biochemical markers

Blood was collected at the end of treatment by cardiac puncture using a syringe (5 ml) with a 23-gauge needle. The animals were then anesthetized using xylazine (10 mg/kg body weight) and ketamine hydrochloride (100 mg/kg body weight) and euthanized by decapitation. Fasting blood cholesterol, TG, LDL, and high-density lipoprotein (HDL) levels in the serum were determined using a commercial assay kit (Huseini et al. 2015).

### Antioxidant and oxidative stress

Catalase, superoxide dismutase, and glutathione peroxidase activities were determined according to Kaddour et al. (2016). Reduced glutathione (GSH), reactive oxygen species (ROS), and lipid peroxidation levels were also determined as described previously (Arutyunyan et al. 2016).

### Histopathological analysis

The rats were anesthetized using xylazine (10 mg/kg body weight) and ketamine hydrochloride (100 mg/kg body weight) and were sacrificed by decapitation. The aorta was then separated from the diaphragm and divided into distal, middle, and proximal segments. The segments were dehydrated using a graded series of alcohol/xylene and then embedded in paraffin. The paraffin blocks were cut into 4 mm^2^ thick sections and stained with hematoxylin and eosin. The stained sections were characterized in terms of the presence of an extracellular lipid core, fatty streaks, and foam cells. The degrees of atherosclerosis and vascular injury were quantitatively determined based on the lesion area using an image analyzer (Kumar et al. 2005).

### Statistical analysis

Data are expressed as mean ± standard deviation (SD). The results were compared using one-way analysis of variance followed by Tukey’s post hoc test. The difference between the control and treated samples was significant at *P* < 0.05.

## Results

Rats feed with 1% HCD exhibited a substantial increase in the levels of LDL, HDL, cholesterol, and TGs. However, rats supplemented with 1, 10, and 100 μM myricitrin showed significant reduction in cholesterol levels of 14.9%, 32.3%, and 62.3%, respectively (Table 1, *P* < 0.05). Rats feed with 1% HCD showed a substantial decrease in HDL levels. However, the rats given 1, 10, and 100 μM myricitrin showed significant increase in HDL levels of 21.4%, 164.3%, and 316.7%, respectively (Table 1, *P *< 0.05).

**Table 1:** Effect of myricitrin on fasting lipid profile and aortic wall area percent (mm^2^) after 45 days of high cholesterol diet (HCD) fed rats treated with myricitrin at dosages of 1, 10 and 100 μM

Groups	Cholesterol (mg/dl)	HDL (mg/dl	LDL (mg/dl)	TG (mg/dl)	Aortic wall area percent (mm^2^)	Percent of aortic wall area changes
Sham (normal diet)	37.2 ± 1.6	19.5 ± 1.1	10.9 ± 0.7	55.6 ± 3.6	0.205 ± 0.017	
Control (1% HCD)	1134.5 ± 25.3***	4.2 ± 0.1***	957.3 ± 15.4***	97.5 ± 6.2**	0.298 ± 0.019*	14.5% ↑ Compared with sham
1 μM of myricitrin + 1% of HCD	965.2 ± 17.2^#^	5.1 ± 0.1^#^	916.6 ± 17.1^#^	92.3 ± 5.8	0.291 ± 0.021	2.3% ↓ Compared with control
10 μM of myricitrin + 1% of HCD	711.8 ± 16.4^##^	11.1 ± 1.1^#^	651.7 ± 11.5^#^	81.7 ± 5.6	0.286 0.015^#^	4% ↓ Compared with control
100 μM of myricitrin + 1% of HCD	427.1 ± 11.2^###^	17.5 ± 1.2^###^	213.5 ± 8.6^###^	73.4 ± 4.4^#^	0.216 0.011^#^	27.5% ↓ Compared with control
Positive control (10 mg/kg body weight of atorvastatine)	213.4 ± 7.8^###^	18.5 ± 1.3^###^	327.8 ± 7.2^###^	76.5 ± 3.7^#^	0.272 0.016^#^	8.7% ↓ Compared with control

Values are expressed as mean with standard deviations (SD). *P* < 0.05 was considered as statistically significant

*** *P* < 0.001 vs. sham group

^#^*P* < 0.05, ^##^ *P* < 0.01 and ^###^ *P* < 0.01 vs. control group

Rats feed with 1% HCD showed a substantial increase in LDL levels. However, the rats supplemented with 1, 10, and 100 μM myricitrin showed significant reduction in LDL levels of 4.3%, 31.9%, and 77.7%, respectively (Table 1, *P* < 0.05). Rats feed with 1% HCD showed increased in TG levels. However, 1, 10, and 100 μM myricitrin exhibited reduction in TG levels of 5.3%, 16.2%, and 24.7%, respectively (Table 1, *P* < 0.05). Rats feed with 1% HCD showed some degree of atherogenesis compared with sham rats. However, 1, 10, and 100 μM myricitrin resulted in reductions in atherogenesis of 2.3%, 4%, and 27.5%, respectively (Table 1, *P *< 0.05).

**Fig. 1:**
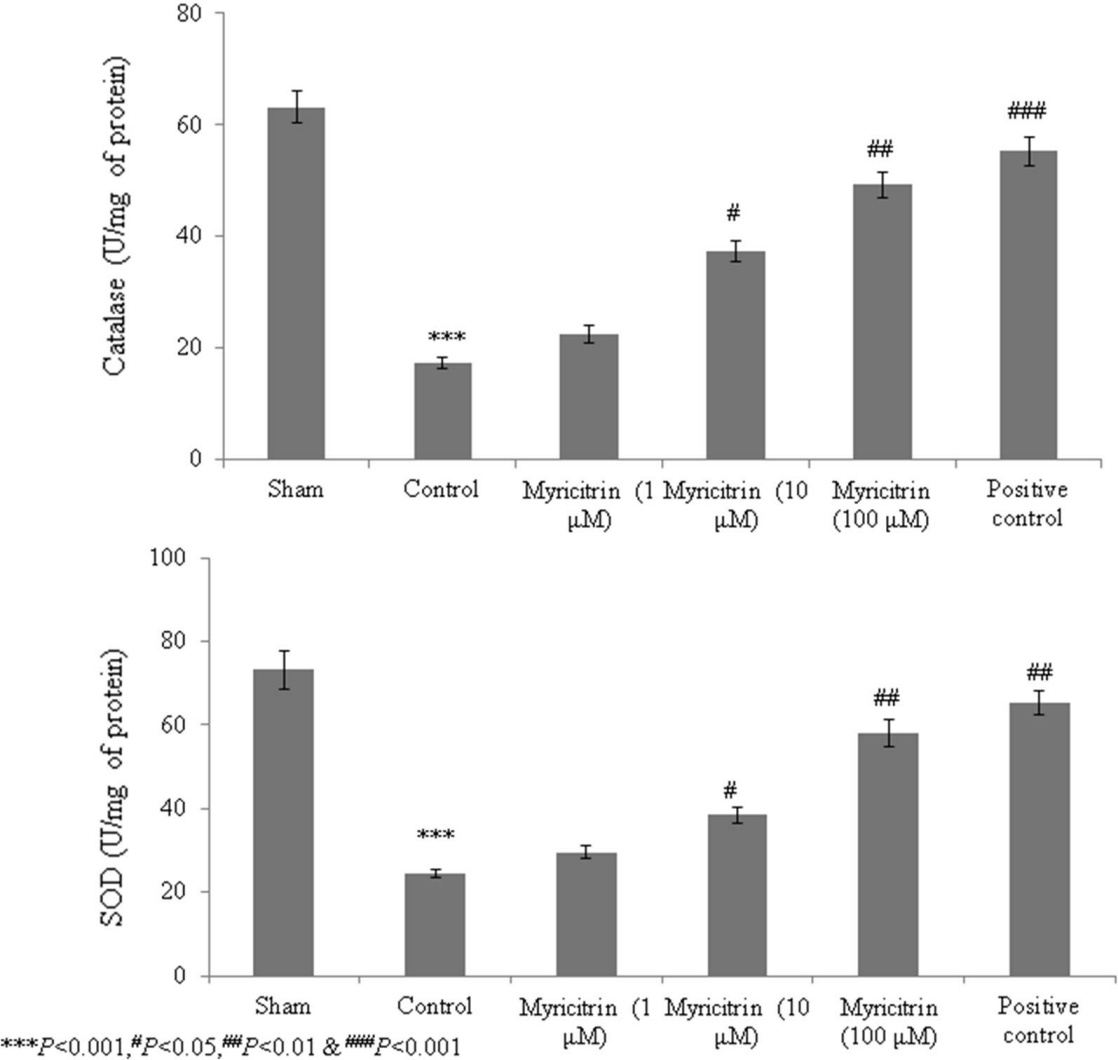
The effect of myricitrin on catalase and superoxide dismutase activities in high-cholesterol diet (HCD)-fed rats. The rats were divided into the following groups: sham (standard food), control (1% HCD), 1 μM myricitrin + 1% HCD, 10 μM myricitrin + 1% HCD, 100 μM myricitrin + 1% HCD, and the positive control (10 mg/kg body weight atorvastatin). Each group contained six rats. Myricitrin was given to the rats via oral gavage for 45 consecutive days. ^***^P < 0.001 compared with the sham group; ^#^*P *< 0.05, ^##^*P *< 0.01, and ^###^*P *< 0.001 compared with control rats

**Fig. 2:**
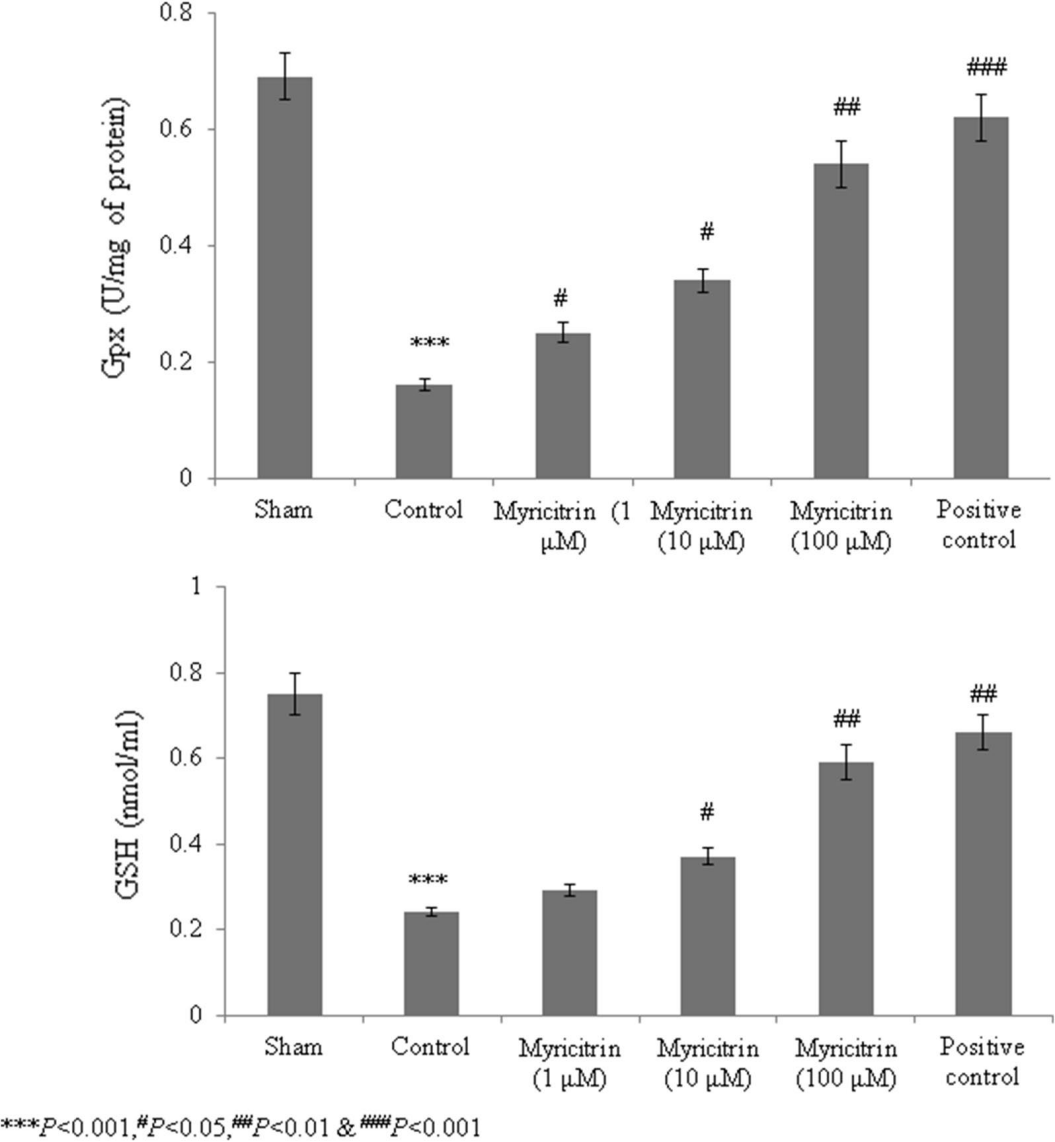
The effect of myricitrin on glutathione peroxidase activity and GSH levels in high-cholesterol diet (HCD)-fed rats. The rats were divided into the following groups: sham (standard food), control (1% HCD), 1 μM myricitrin + 1% HCD, 10 μM myricitrin + 1% HCD, 100 μM myricitrin + 1% HCD, and the positive control (10 mg/kg body weight atorvastatin). Each group contained six rats. Myricitrin was given to the rats via oral gavage for 45 consecutive days. ^***^P < 0.001 compared with the sham group; ^#^*P *< 0.05, ^##^*P *< 0.01, and ^###^*P *< 0.001 compared with control rats

**Fig. 3:**
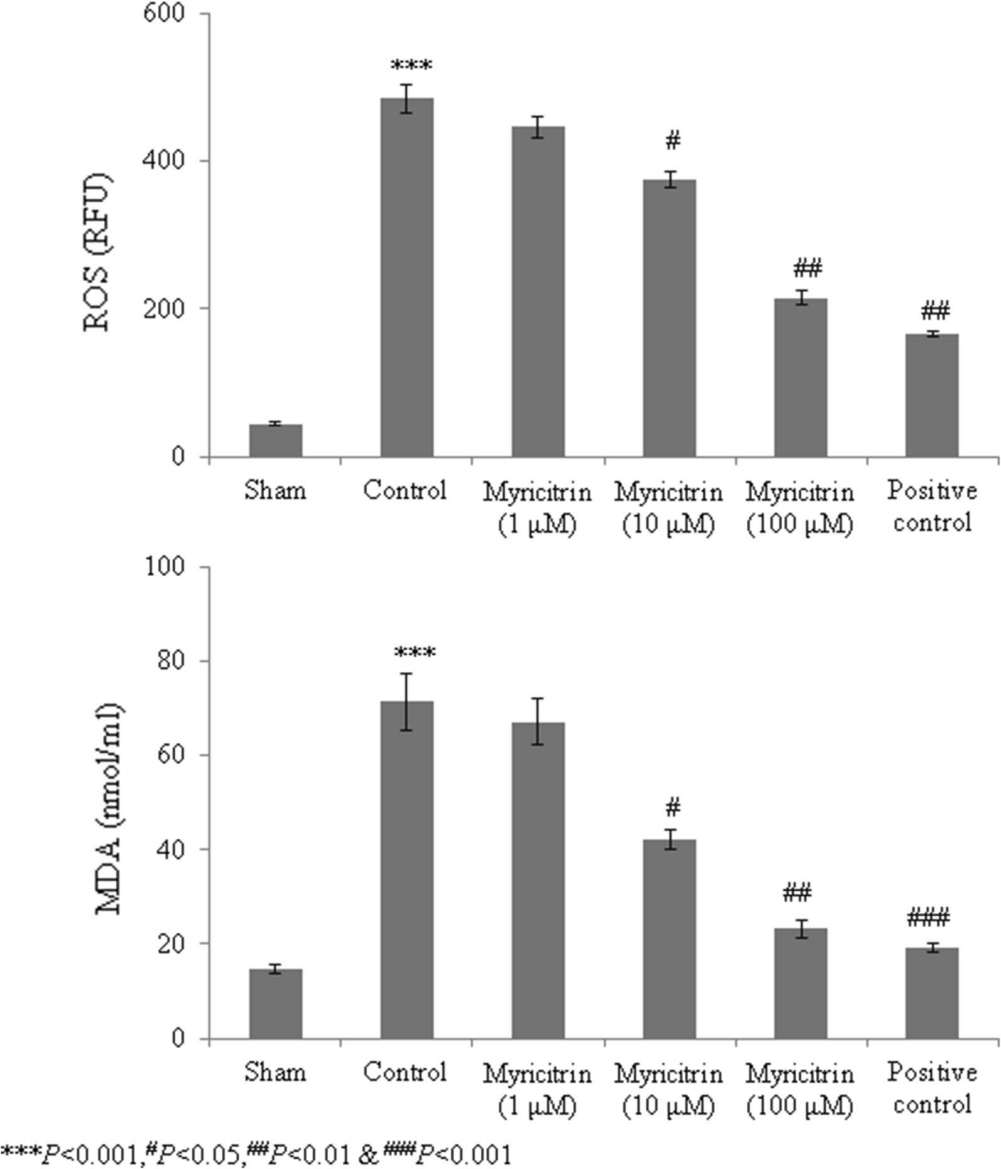
The effect of myricitrin on reactive oxygen species and malondialdehyde levels in high-cholesterol diet (HCD)-fed rats. The rats were divided into the following groups: sham (standard food), control (1% HCD), 1 μM myricitrin + 1% HCD, 10 μM myricitrin + 1% HCD, 100 μM myricitrin + 1% HCD, and the positive control (10 mg/kg body weight atorvastatin). Each group contained six rats. Myricitrin was given to the rats via oral gavage for 45 consecutive days. ^***^P < 0.001 compared with the sham group; ^#^*P *< 0.05, ^##^*P *< 0.01, and ^###^*P *< 0.001 compared with control rats

**Fig. 4:**
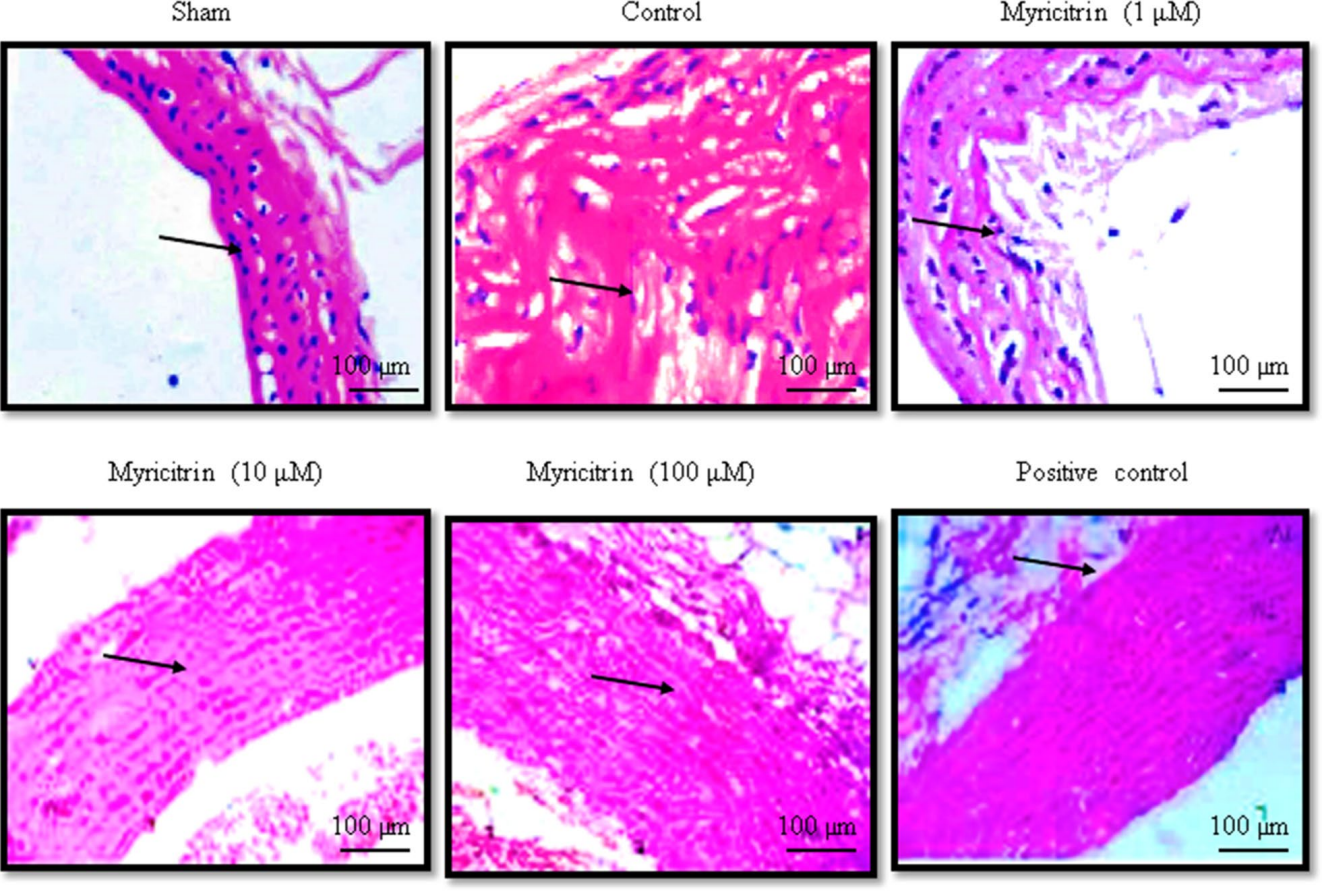
The effect of myricitrin on morphological changes in high-cholesterol diet (HCD)-fed rats. The rats were divided into the following groups: sham (standard food), control (1% HCD), 1 μM myricitrin + 1% HCD, 10 μM myricitrin + 1% HCD, 100 μM myricitrin + 1% HCD, and the positive control (10 mg/kg body weight atorvastatin). Each group contained six rats. Myricitrin was given to the rats via oral gavage for 45 consecutive days. Magnification: ×100

Catalase, superoxide dismutase, glutathione peroxidase, and GSH levels were substantially reduced in HCD-fed rats compared with the sham rats. However, rats given 100 μM myricitrin showed increased levels of these molecules of > 50% (Figs. 1 and 2, *P* < 0.05). ROS and malondialdehyde levels were substantially increased in HCD-fed rats compared with sham rats. However, the rats supplemented with 1, 10, and 100 μM myricitrin showed significant reductions in ROS levels of 8%, 22.6%, and 55.7% and in malondialdehyde levels of 5.9%, 41%, and 67.5%, respectively (Fig. 3, *P* < 0.05). The aortic cell wall area was significantly increased by 14.5% in HCD-fed rats compared with sham rats but was significantly reduced by 1, 10, and 100 μM myricitrin by 4%, 8.3%, and 17.3%, respectively (Fig. 4; Table 1, *P* < 0.05).

## Discussion

We showed the anti-atherosclerotic potential of myricitrin in hypercholesterolemic rats. Our results suggest that myricitrin treatment reduces the levels of LDL, TG and total cholesterol and the role of aortic atherosclerosis in hypercholesterolemic rats. Studies have shown that atherosclerosis is a complex disease process due to the interactions between inflammation and perturbations (van Diepen et al. 2013), and inflammation and dyslipidemia are the primary mechanisms involved in the pathophysiology of atherosclerosis (Singh et al. 2002). Increased levels of total cholesterol, plasma TGs, and LDL cholesterol are characteristic features of hyperlipidemia (Tonstad and Despres 2011), and obesity, dyslipidemia, and hypertension trigger several inflammatory events, which cause foam cell formation and monocyte recruitment to atherosclerotic lesions (Libby et al. 2002). The possible underlying mechanism of inhibition of aortic atherosclerotic progression by myricitrin may not be due solely to its lipid-lowering potential, because LDL, TG, and total cholesterol levels were higher in HCD-fed rats, compared with sham rats. In our study, 100 µM of myricitrin showed maximum protective effect against higher LDL, TG, and total cholesterol levels.

Atherosclerotic plaque development was found to require increased levels of LDL and total cholesterol and oxidation of LDL-cholesterol (Badimon and Vilahur 2012), and studies have shown the anxiolytic and antinociceptive effects of myricitrin (Pereira et al. 2011). Sun et al. (2013) reported the protective effect of myricitrin against the vascular endothelial cell apoptosis induced by oxidative stress and the inhibitory effects of myricitrin against the endothelial damage and early atherosclerosis induced by oxidative stress in ApoE^−/−^ mice. Qin et al. (2015) reported the protective effect of myricitrin against oxidized LDL-induced endothelial cell apoptosis, and also reported its possible therapeutic activities against cardiovascular diseases. Inhibition of lipid peroxidation and oxidative stress was suggested to have a beneficial impact on preventing atherogenesis (Heinecke 1998). Several studies showed that the flavonoids inhibit the oxidation of LDL-cholesterol, protect the vascular endothelium against oxidative injury, and improve dyslipidemia (Martin and Andriantsitohaina 2002; Loke et al. 2010; Mulvihill and Huff 2010). To the best of our knowledge, this is the first report describing the anti-atherosclerotic and hypolipidemic effects of myricitrin in hypercholesterolemic rats.

## Data Availability

Corresponding author could provide the all experimental data on valid request.
